# Arabic version of the simplified diabetes knowledge scale: psychometric and linguistic validation

**DOI:** 10.11604/pamj.2022.41.346.33522

**Published:** 2022-04-28

**Authors:** Maali Haoues, Chekib Zedini, Molka Chadli-Chaieb

**Affiliations:** 1 Faculty of Medicine Ibn El Jazzar of Sousse, University of Sousse, Sousse, Tunisia,; 2 Department of Community Medicine, Faculty of Medicine Ibn El Jazzar of Sousse, University of Sousse, Sousse, Tunisia,; 3 Department of Endocrinology and Metabolic Diseases, Farhat Hached University Hospital, Sousse, Tunisia

**Keywords:** Diabetes, knowledge, translation, validation

## Abstract

**Introduction:**

the simplified diabetes knowledge scale is used to obtain a general assessment of diabetic´s knowledge about diabetes and its care. For clinical and methodological purposes, it was relevant and necessary to develop an Arabic version of this instrument. Thus, the aim of this study was to translate and validate the simplified diabetes knowledge scale (SDKS) into Arabic to measure the knowledge of Arabic-speaking diabetics.

**Methods:**

a methodological validation study of the simplified diabetes knowledge scale, following the guidelines of Vallerand was carried out. A convenience sample of diabetics followed in eight basic health centers in Sousse region and in Farhat Hached and Sahloul University Hospitals was recruited. An Arabic questionnaire including the demographic and clinical data of the diabetic and the final experimental version of the simplified diabetes knowledge scale was used.

**Results:**

a sample of 333 diabetics was recruited. Content validity of the final experimental version was 0.94. Reliability assessed by Cronbach´s alpha coefficient (0.812), by test-retest correlation coefficient (> 0.60) and by internal consistency after deletion of each item (from 0.788 to 0.816) were acceptable except items 19 and 20 which had to be reformulated. Construct validity analysis identified that three items among the 20 ones (12, 17 and 20) required reformulation. Inter-item correlation matrix showed that the majority of items were not correlated with each other. Validation process was ended by establishing standards table.

**Conclusion:**

this study showed the Arabic version of the simplified diabetes knowledge scale had good validity and reliability.

## Introduction

Tunisia is experiencing an explosion of diabetes mellitus pandemic with a national prevalence of about 10.2% in 2019 [1] and estimated at 11.7% in 2030 [2]. The evolution of the disease concerns primarily type 2 diabetes. Type 1 is less common [1,2]. Aging populations and their increased exposure to diabetes and cardiovascular disease risk factors such as smoking, overweight and obesity, physical inactivity, stress, and inadequate diet favor this meteoric rise [3-9]. The best weapons to fight this increasing are information and education [10,11]. To achieve better control of their health and physical well-being, diabetics will need to acquire knowledge about their disease [11]. The majority of instruments measuring diabetes knowledge have been designed and validated in English for the American and British populations [12,13]. Few have been validated in Arabic [14,15]. The simplified diabetes knowledge scale (SDKS) is used to obtain a general assessment of diabetic´s knowledge about diabetes and its care [16]. For clinical and methodological purposes, it was relevant and necessary to develop an Arabic version of this instrument. Thus, the aim of this study was to translate and validate the SDKS into Arabic to measure the knowledge of Arabic-speaking diabetics.

## Methods

**Study design, duration and setting:** a methodological study was undertaken to validate the SDKS into Arabic. The adopted approach was based on the cross-cultural validation technique described by Vallerand [17]. The study started on March 8, 2019 and ended on July 1, 2020. It was of interest to the services that cared for diabetics at the two university hospitals Sahloul and Farhat Hached and eight basic health centers in the Sousse region (Tunisia).

**Study participants:** patients with diabetes type 1 and type 2, meeting the following inclusion criteria, were recruited: diabetic aged 18 years and older, whose disease has been evolving for at least one year and able to read and understand an Arabic language newspaper. Any diabetic with cognitive impairment detected by a score greater than or equal to 14 on the Mini-Mental State Examination in its Tunisian version [18] was excluded from the study.

**Study sampling:** diabetics were collected following convenience sampling, which consists of choosing arbitrarily, people according to their accessibility and availability in a specific place and at a specific time.

**Sample size:** the minimum number of participants estimated to validate the SDKS was set at 200 diabetics, according to the rule, which stipulates that the number of participants required for the validation of an instrument depend on the number of its items, which is ten at least to evaluate each item [19].

**Data collection:** a developed self- administered questionnaire containing two sections was used. The first section was designed to collect diabetic's demographic and clinical data (age, sex, type of diabetes, duration of diabetes and diabetes treatment). The second section contained the SDKS, a questionnaire that contains 20 items, 18 are general and two are specific to insulin-treated diabetics. The items address diet, exercise, Glycated Hemoglobin (HbA1c), foot care, regular follow-up and informations about diabetes complications. Responses are in a “True/False/Don´t Know” format. The purpose of this questionnaire is to obtain a general assessment of diabetic's knowledge of diabetes and its care. The clarity of the items and the simplicity of the response method provided an opportunity for people with limited education to participate [16]. The proportion of correct answers represents SDKS score. The maximum score is 18 for non-insulin-treated diabetics and 20 for insulin-treated ones. Diabetics who answered more than 65% of the questions correctly (i.e. 13/20 or 12/18 correct answers) were considered to have good knowledge of diabetes mellitus [16].

**Translation and validation process:** the methodology was carried out in seven steps based on the cross-cultural validation technique described by Vallerand [17]: a. Preparation of preliminary versions by parallel reverse translation, i.e. two translations from English to Arabic and two others from Arabic to English. b. Evaluation of the preliminary versions and preparation of an experimental one using the Delphi method [20], by a panel of 13 experts in diabetes, translation and languages. This panel included the author of the work, three experts in research and cross-cultural validation, three specialists in Diabetology, the four translators, an English professor familiar with medical terms and an Arabic professor. c. A pre-test of the experimental version was carried out with 30 Tunisian diabetics. The same panel made modifications and reformulations, after approval, in order to establish a final experimental version. These diabetics were excluded from the subsequent statistical analysis. d. The second panel of six experts, different from the first one composed of four specialists in Diabetology and two epidemiologists calculated the content validity index (CVI) to judge the clarity and relevance of the items' statements and determined content validity of the final experimental version using the Delphi method [20]. e. The final experimental version of the questionnaire was administrated to a group of 333 diabetics. Reliability was measured by evaluating the internal consistency (calculation of the Cronbach's alpha coefficient), calculating the mean and the variance of the questionnaire after reduction of the items (deletion of the items one by one), determining the complete correlation of the corrected items and calculating the Cronbach's alpha of the whole of the items but one. The questionnaire was administered a second time in a random subgroup of the 333 participants, one month apart, to study its temporal stability and measure its reproducibility. One hundred and sixty diabetics gave survey feedback. f. Construct validity was assessed by the correlation between item responses and the questionnaire score and by the inter-item correlation matrix. g. This process was concluded with the establishment of the questionnaire standards.

**Statistical analysis:** statistical study was carried out using SPSS version 21.0 software. Categorical variables were expressed as relative frequency (%). Quantitative variables were summarized by measures of central tendency (mean: M) and dispersion (standard deviation: SD) when they followed the normal distribution or by the median (Med) and interquartile ranges (IIQ: 25th quartile and 75th quartile). The CVI was calculated by dividing the number of statements scoring 3 and 4 by the total number of statements. A CVI equal to or greater than 0.80 indicated acceptable validity [19]. The internal consistency was explored using the Cronbach's alpha formula, for which a value of 0.5 was acceptable and values between 0.70 and 0.85 was desirable [17]. When this coefficient increased with the deletion of an item, it indicated that the item is poorly correlated with other questionnaire items and that it should be excluded or modified [21]. The Pearson r correlation coefficient between test and retest scores, one month apart was interpreted as satisfactory according to Vallerand [17] if it was positive and ≥ 0.60. The intra-class correlation coefficients (ICC) were calculated between test and retest response scores, with a 95% confidence interval, and interpreted as follows: very good if ICC ≥ 0.91; good if 0.90 ≤ ICC ≤ 0.71; moderate if 0.70 ≤ ICC ≤ 0.51; low if 0.50 ≤ ICC ≤ 0.31 and very low if ICC ≤ 0.30 [22]. To measure the reproducibility between test and retest, Cohen´s d coefficient was tested by comparing the mean difference in response scores between test and retest, divided by their standard deviation. A value of 0.2 indicated a “small” effect and was considered to have a satisfactory reproducibility; a value of 0.5 indicated a “Medium” effect and a value of 0.8, a “Large” effect [23]. The construct validity was determined by studying the relationship between items and questionnaire score. It was measured using Pearson's correlation coefficient, a value greater than or equal to 0.4 indicated that the item provided sufficient information on the score [24]. Inter-item correlation values < 0.2 indicated the absence of correlation between items. They were considered acceptable if they varied between 0.3 and 0.7 [24]. A positive correlation indicated a simultaneous variation in the same direction and a negative correlation indicated a simultaneous variation in the opposite direction [24]. Norm table establishment required the determination of the percentile rank, the mean and the standard deviation of the questionnaire score as well as the calculation of Z and T. A T-score of 45 means that one was 1/2 standard deviation below the mean. This corresponds to a z score of -0.5. A value of 69.6 means that one was 1.96 standard deviations above the mean (only 2.5% of diabetics would have a higher score) [24]. The significance level of all the tests was set at p ≤ 5%.

**Ethical consideration:** the Human Research Ethics Committee of the Faculty of Medicine of Sousse has approved this project on July 27, 2020, under the reference CEFMS 54 / 2020. A license was obtained from the Michigan Diabetes Research and Training Center for translation and validation of the original SDKS. Authorizations from the directors and heads of departments of the study sites were also obtained. All participants read and signed a consent form in Arabic, validated by the Ethics Committee. The copyright for the validated Arabic version of the instrument is reserved to the authors of the article and will be provided upon request.

## Results

**Demographic and clinical data of the study population:** three hundred and thirty-three diabetics were included. Their mean age was 51.11 (16.21) years with extremes from 18 to 90 years. The sex ratio was 0.94. Two hundred forty-three participants (73%) had type 2 diabetes and 90 had type 1. The median age of the disease was 7 years with extremes of 1 to 38 years. Insulin was used by 60.7% of them.

**Content validity of the SDKS final experimental version:** experts reviewed each item of the final experimental version for relevance, accuracy, and representativeness. Their judgment varied from item to item. The CVI for the questionnaire was 0.94.

**Reliability of the SDKS final experimental version:** internal consistency, assessed by Cronbach´s alpha, calculated from the questionnaire responses of 333 diabetics, was 0.812. One hundred and sixty diabetics among the 333 ones responded to the final experimental version of DHP-18, twice, 30 days apart. Test- Retest correlation coefficient of the questionnaire indicated a satisfactory temporal stability at one month (0.955). Cohen´s d coefficient reflected a “small” associated effect (-0.111) and the ICC was close to one (0.977) indicating similarity of responses within the same group after the 30-day interval. Internal consistency after deletion of each item ranged from 0.788 to 0.816. The removal of items 3, 4, 8, and 9 reduced the internal consistency of the questionnaire (between 0.788 and 0.794) (Table 1). In return, the removal of items 11 and 19 did not modify it (Cronbach´s alpha value (0.812)) (Table 1). Deleting item 20 increased the value of Cronbach´s alpha from 0.807 to 0.816 (Table 1) which reduced the quality of the questionnaire and required its elimination or reformulation. Theses finding were confirmed by the interpretation of the complete correlation coefficients of the corrected items (Table 1). They ranged from 0.543 to 0.695 for items 3, 4, 8 and 9. Item 20 had a complete corrected correlation coefficient of 0.05. Item 19 had a value of 0.177 (<0.2) with a stable Cronbach´s alpha value when deleted (0.812). Conversely, item 11 had an acceptable coefficient (0.217) although its deletion did not change the Cronbach´s alpha value (0.812). These results implied the need to reword or remove it (Table 1).

**Table 1:** reliability of the final experimental version of SDKS after removal of each item (n=333)

Item	Average of the questionnaire after deletion of the item	Variance of the questionnaire after deletion of the item	Complete correlation of corrected items	Cronbach's Alpha if the item is deleted
1	23.90	45.416	0.218	0.811
2	14.19	43.146	0.396	0.803
3	24.72	41.441	0.695	0.789
4	24.63	41.006	0.677	0.788
5	24.06	43.189	0.376	0.804
6	23.86	43.842	0.357	0.805
7	24.16	42.490	0.441	0.800
8	24.32	40.692	0.543	0.794
9	24.11	40.147	0.591	0.790
10	24.25	42.912	0.406	0.802
11	23.78	45.250	0.217	0.812
12	24.28	43.411	0.276	0.811
13	24.13	42.878	0.393	0.803
14	24.94	45.294	0.406	0.802
15	24.87	42.716	0.374	0.804
16	23.92	43.726	0.303	0.808
17	24.49	44.083	0.336	0.806
18	24.31	44.403	0.252	0.811
19	23.77	46.030	0.177	0.812
20	23.75	46.949	0.055	0.816

**Construct validity of the SDKS final experimental version:** construct validity was measured by assessing the degree of association between each item and the SDKS score. Pearson test results showed that all correlation coefficients were significant at the 0.01 level excluding items 12 and 20 (Table 2). Items 12, 17, and 20 had a very low item-global score correlation (<0.2) whereas items 1, 3, 4, 8, 9, and 13 had satisfactory correlations (>0.4) (Table 2). All item-global score correlation coefficients were positive, indicating simultaneous variation in the same direction. Only item 20 had a negative correlation indicating simultaneous variation in the opposite direction (Table 2). After reformulation of the above items, the questionnaire was administered again to 10 new diabetics to check for clarity. The CVI was recalculated. The new value was 0.96, higher than the initial value of 0.94. Inter-item correlation matrix showed that the majority of items were not correlated with each other (correlation coefficients < 0.2). Only a few items were slightly and positively correlated with each other (Table 3).

**Table 2:** SDKS item-score correlation study (n=333)

Items SDKS score	Item-Score correlation
**SDKS1**	0.485**
**SDKS2**	0.229**
**SDKS3**	0.533**
**SDKS4**	0.467**
**SDKS5**	0.213**
**SDKS6**	0.327**
**SDKS7**	0.321**
**SDKS8**	0.613**
**SDKS9**	0.621**
**SDKS10**	0.260**
**SDKS11**	0.263**
**SDKS12**	0.085
**SDKS13**	0.523**
**SDKS14**	0.447**
**SDKS15**	0.338**
**SDKS16**	0.236**
**SDKS17**	0.189**
**SDKS18**	0.343**
**SDKS19**	0.396**
**SDKS20**	-0.25

** The correlation is significant

**Table 3:** SDKS inter items correlation matrix

	SDKS1	SDKS2	SDKS3	SDKS4	SDKS5	SDKS6	SDKS7	SDKS8	SDKS9	SDKS10	SDKS11	SDKS12	SDKS13	SDKS14	SDKS15	SDKS16	SDKS17	SDKS18	SDKS19	SDKS20
**SDKS1**	1.000																			
**SDKS2**	.160	1.000																		
**SDKS3**	.130	.189	1.000																	
**SDKS4**	.073	.216	.754	1.000																
**SDKS5**	.172	.419	.315	.298	1.000															
**SDKS6**	.075	.105	.295	.327	.116	1.000														
																				
**SDKS7**	.107	.157	.348	.326	.230	.343	1.000													
**SDKS8**	.038	.252	.556	.579	.247	.263	.298	1.000												
**SDKS9**	.018	.298	.582	.563	.318	.222	.440	.590	1.000											
**SDKS10**	-.163	.211	.383	.336	.167	330	.281	.261	.525	1.000										
**SDKS11**	.192	.124	.088	.127	.125	.182	.028	.005	-.013	.168	1.000									
**SDKS12**	-.095	.154	.266	.252	.202	.065	.207	.119	.180	.182	.014	1.000								
**SDKS13**	.224	.228	.335	.373	.184	.112	.131	.338	.290	.174	.071	.161	1.000							
**SDKS14**	.124	.139	.386	.428	.085	-.093	.133	.442	.324	.032	.030	.177	.185	1.000						
**SDKS15**	.033	.122	.394	.357	.054	.020	.145	.405	.273	.162	-.081	.187	.202	.572	1.000					
**SDKS16**	.119	.207	.208	.177	.102	.237	.214	-.017	.132	.300	.311	.081	.027	.132	.111	1.000				
**SDKS17**	.035	.196	.251	.184	.119	.183	.206	.133	.162	.142	.182	.095	-.007	.348	.219	.253	1.000			
**SDKS18**	.148	.066	.264	.218	.039	.240	.103	.068	.084	.118	.226	.151	.144	.022	-.045	.109	.232	1.000		
**SDKS19**	.688	.183	.028	.010	.127	.071	.085	-.022	.016	-.109	.189	-.109	.225	.053	.040	.046	.037	.133	1.000	
**SDKS20**	.236	.072	-.043	.017	.008	.159	.037	-.031	-.120	-.145	.206	.075	.110	-.127	-.057	.054	-.056	.110	.168	1.000

**SDKS norm setting:** Z scores ranged from -1.597 to 1.767. T scores drew a Gaussian curve and ranged from 34.031 to 67.677 (Table 4). The original shape of the distribution was preserved and the interval between T units was equidistant across the entire questionnaire. In addition, all of percentile ranks ≤ 30 had a T score ≤ 45, meaning that the responses were 1/2 standard deviation below the mean and corresponded to a Z value ≤ -0.5 (Table 4).

**Table 4:** SDKS standards table (n = 333)

Percentiles	Z Score	T Score
5	-1.597	34.031
10	-1.400	35.997
20	-0.854	41.459
25	-0.608	43.917
30	-0.581	44.190
40	-0.308	46.921
50	-0.348	49.652
60	0.238	52.383
70	0.293	52.929
75	0.511	55.114
80	0.785	57.845
90	1.495	64.946
95	1.767	67.677

**Diabetics knowledge level:** the majority of diabetics (81.7%) had a low level of knowledge about their disease (mean diabetes knowledge score measured by the SDKS ≤ 65%). Table 5 describes the responses to each item in the questionnaire.

**Table 5:** description of diabetics knowledge level out their disease (n=333)

Items	I don ’t Know		False		True	
	n	%	n	%	n	%
SDKS1	21	6.3	86	25.8	226	67.9
SDKS2	47	14.1	110	33.0	176	52.9
SDKS3	93	27.9	202	60.7	38	11.4
SDKS4	90	27.0	183	55.0	60	18.0
SDKS5	42	12.6	82	24.6	209	62.8
SDKS6	34	10.2	41	12.3	258	77.5
SDKS7	57	17.1	111	33.3	165	49.6
SDKS8	87	26.1	77	23.1	169	50.8
SDKS9	77	23.1	28	8.4	228	68.5
SDKS10	64	19.2	120	36.0	149	44.8
SDKS11	33	9.9	33	9.9	267	80.2
SDKS12	111	33.3	40	12.0	182	54.7
SDKS13	52	15.6	66	19.8	215	64.6
SDKS14	204	61.3	31	9.3	98	29.4
SDKS15	196	58.9	60	18.0	77	23.1
SDKS16	79	23.7	18	5.4	236	70.9
SDKS17 (n=203)	44	21.7	113	55.6	46	22.7
SDKS18 (n=203)	42	20.7	81	39.9	80	39.4
SDKS19	12	3.6	56	16.8	265	79.6
SDKS20	4	1.2	82	24.6	247	74.2

**Funding:** this research did not receive any specific grant from funding agencies in the public, commercial, or not-for-profit sectors.

## Discussion

Our study aimed to translate and validate the Arabic version of the SDKS. Based on our study results, the Arabic version of SDKS is a validated tool that can be used in the Arabic-speaking population with type 1 and type 2 diabetes. Findings provided a valid and a reliable Arabic version of the SDKS. They showed an acceptable content and construct validity, internal consistency, temporal stability and reproducibility of the questionnaire.

Before undertaking the present study, a bibliographical research was achieved to inventory instruments available in Arabic and English measuring diabetes Knowledge. None of the Arabic instruments was retained because they were not validated [16,25]. In English, the SDKS met the criteria of content, conciseness, simplicity, reliability and validity [16]. However, neither of its two Arabic translations met validation process [26,27]. Therefore, developing a translated version of this tool and validating it according to Vallerand's cross-cultural translation guidelines was considered useful [17]. Some steps did not take place because the study instrument is not a psychometric scale.

The CVI of the SDKS experimental version (0.94) showed that the items accurately measured the concepts explored. Internal consistency, assessed by Cronbach´s alpha, was 0.812. According to the standards established by Vallerand [17], this value is satisfactory. This result is similar to that reported by Collins GS *et al*. [16], during the development of the SDKS. The internal consistency, calculated from the questionnaire responses was 0.71 [16]. Khunkaew S *et al*. [28] for their part found a satisfactory internal consistency value of 0.79. Temporal stability (test-retest reliability), evaluated from the responses of 160 diabetics to SDKS final experimental version, on two occasions, one month apart, was satisfactory indicating a similarity of responses within the same group after the 30-day interval.

Internal consistency after deletion of each item, supplemented by the calculation of the complete correlation coefficients of the corrected items, assessed the respective importance of each item and considered the reformulation of the items whose values in statistical tests deviated from the desired values. In our study, items 19 and 20 had complete correlation coefficient of corrected items of 0.171 and 0.05 respectively, with Cronbach´s alpha values after removing each item equal to 0.812 and 0.816 respectively, requiring their rewording (Table 1). Our results agree with those of Collins GS *et al*. [16]. Cronbach´s alpha values after deletion of each SDKS item were lower than questionnaire Cronbach's alpha value (0.71). They ranged from 0.57 to 0.62 [16]. Complete correlation coefficients of the corrected items were > 0.2, except for items 7, 8 and 20, to which the authors of the study made rewordings [16]. Khunkaew S *et al*. [28] adopted the same strategy. SDKS internal consistency after deletion of each item found values above 0.7, ranging from 0.775 to 0.786. The deletion of each item reduced the value of the questionnaire internal consistency to 0.79 [28]. Complete correlation coefficients of the corrected items were > 0.2, excluding item number 19, which required rewording [28].

Construct validity retained 17 items and identified three with low item-global score correlation coefficients: item 20 having been previously corrected and items 12 and 17 were reformulated. This analysis improved the overall CVI of the questionnaire from 0.94 to 0.96. Collins GS *et al*. [16] also analyzed the degree of association between each item and SDKS score. Correlation coefficients were acceptable, ranging from 0.26 to 0.58 and items were unchanged. In our study, inter-item correlation matrix showed that the majority of items were not correlated with each other (< 0.2) (Table 3). Since the SDKS is a non-psychometric questionnaire and does not include theory-based dimensions, this result was acceptable. Although each item contains information independent of the others, they all measure the same construct (knowledge about diabetes). In the study of Khunkaew S *et al*. [28] inter-item correlation matrix had correlation values ranging from 0.03 to 0.49. The majority of the items were not correlated with each other with correlation coefficients < 0.2.

The study is to some degree limited by the non-random sampling technique used to recruit study participants although it is the most widespread to meet the objective of this research. Despite this limitation, the psychometric validation study of SDKS in Arabic resulted in the production of a reliable and valid translated version.

## Conclusion

This study showed the Arabic version of the SDKS had good validity and reliability. This tool needs to be tested on a larger number of diabetics to confirm the reproducibility of its results.

### What is known about this topic

- Good knowledge about diabetes is associated with favorable outcomes among patients with diabetes;
- Few of instruments measuring diabetes knowledge have been validated in Arabic.

### What this study adds

- A valid and reliable Arabic instrument was produced, for assessing diabetes knowledge;
- This instrument could be used in all Arabic-speaking countries.
